# Immunity Against Bacterial Infection of the Central Nervous System: An Astrocyte Perspective

**DOI:** 10.3389/fnmol.2019.00057

**Published:** 2019-03-06

**Authors:** Sohair Geyer, Muazzam Jacobs, Nai-Jen Hsu

**Affiliations:** ^1^Division of Immunology, Department of Pathology, Institute of Infectious Disease and Molecular Medicine, Faculty of Health Sciences, University of Cape Town, Cape Town, South Africa; ^2^National Health Laboratory Service, Johannesburg, South Africa; ^3^Immunology of Infectious Disease Research Unit, South African Medical Research Council, Cape Town, South Africa

**Keywords:** astrocyte, T cell, bacteria, infection, neuroinflammation, central nervous system

## Abstract

Bacterial infection of the central nervous system (CNS) is a severe and life-threatening condition with high mortality, and it may lead to permanent neurological deficits in survivors. Increasing evidence indicates that astrocytes, as the most abundant CNS glial cell population, regulate innate and adaptive immune responses in the CNS under pathological conditions in addition to their role in the maintenance of CNS homeostasis and neuronal function. Following antigen recognition, astrocytes participate in the initiation of innate immune responses, and prompt an adaptive immune response to recruit peripheral immune cells. Investigations have been conducted to understand the immunological role of astrocytes in CNS disease and injury, however, their part in bacterial infections of the CNS has not been fully evaluated. A better understanding will permit the identification of successful therapeutic targets for an improved prognosis and disease outcome.

## Graphical Abstract

Astrocyte plays an important role in the CNS innate and adaptive immune responses to bacterial pathogens.

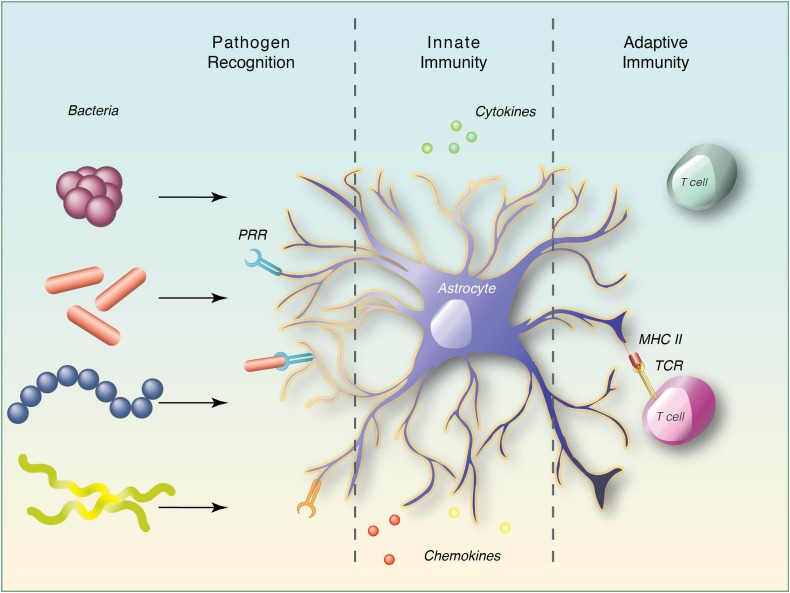

## Introduction

Invasion of the CNS by infectious agents is a major global healthcare concern, and is associated with high morbidity and mortality (John et al., 2015; Robertson et al., 2018). Clinically, the primary classification of CNS infections is based on the affected anatomical regions such as meningitis, encephalitis, and myelitis. Anatomically, the CNS is uniquely compartmentalized in disparate regions where barriers established by endothelial, epithelial and the glial limitans effectively controls the access of the immune system to the CNS (Engelhardt et al., 2017). This evolutionary adaptation protecting it from damaging immune-mediated inflammation has given the perception of the CNS as an immune-privileged site. However, the mechanisms of immune privilege are redefined by the discovery of a lymphatic system within the meninges which may represent a credible path for circulating immune cells to access and patrol meningeal compartments (Louveau et al., 2015). Compartmentalization of immune privilege allows areas of the CNS to be under constant surveillance, enabling resident cells to respond quickly and effectively to pathogenic challenges, simultaneously recruiting cells of the peripheral adaptive immune system; whilst the tight regulation of entry into the CNS parenchyma maintains tissue homeostasis (Galea et al., 2007; Engelhardt et al., 2017).

Despite the presence of effective barriers, various pathogens such as viruses, bacteria, fungi, protozoa, and parasites can disrupt the blood brain barrier (BBB) that may often have chronic implications or prove to be fatal. Viral infections of the CNS have been discussed extensively in current literature, with emphasis on disease progression, especially in immune-compromised individuals. However, bacterial infection of the CNS is potentially more threatening in terms of disease severity, particularly in developing countries where bacterial meningitis is a leading cause of severe neurological sequelae and high mortality. Among a few of the bacteria involved in CNS infections are *Listeria monocytogenes, Borrelia burgdorferi, Neisseria meningitidis, Streptococcus pneumoniae, Staphylococcus aureus, Haemophilus influenzae, Mycobacterium* and *Brucella* species (Drevets et al., 2004; John et al., 2015; Robertson et al., 2018). Bacterial infections of the CNS, which disproportionally affect developing countries, are, however, not as actively investigated.

Astrocytes, from the literal Greek “*star cell*,” also known as astroglia are resident cells of the CNS. Astrocytes are of neuro-ectodermal origin and are strategically located at the interface between blood vessels and the brain parenchyma where they have a dynamic role in maintaining CNS homeostasis, particularly to regulate synapse formation and support neuronal function in both healthy and injured brain (Eroglu and Barres, 2010; Zamanian et al., 2012; Zhang et al., 2014; Allen and Lyons, 2018). Another CNS-resident glial type, microglia are arguably the prime immune-effector cells of the CNS that initiate innate immune responses through antigen presentation and facilitate subsequent neuroinflammation. Microglial function is regulated by different cell types either in a paracrine manner or through direct interaction, amongst which astrocytes play a critical role (Ransohoff and Cardona, 2010). Activation of microglia and astrocytes is a sensitive indication of changes in the CNS microenvironment. Glial cells are able to elicit an innate immune response through recognition of highly conserved motifs, generally referred to as pathogen-associated molecular patterns (PAMPs) by different classes of PRRs such as TLRs, nucleotide-binding oligomerization domain (NOD)-like receptors, scavenger, complement, and mannose receptors (Jack et al., 2005; Braun et al., 2011; Liddelow et al., 2017). Bacterial products such as LPS and bacterial DNA provide adequate stimuli to activate astrocytes. Interestingly, astrocytes become reactive upon stimulation and contribute to brain inflammation through the release of specific cytokines and chemokines (Table 1). Ancillary to facilitating innate immune responses, reactive astrocytes express MHC class II and co-stimulatory molecules, such as CD80 (B7-1) and CD86 (B7-2), that may contribute to T cell activation and integrate communication between resident CNS cells and hematopoietic cells, driving an adaptive immune response (Carpentier et al., 2005). The involvement of astrocytes in the devastating outcomes of CNS bacterial infection is likely a consequence of both the loss of homeostatic functions and gain of neurotoxic effects. The current review therefore evaluates the potential role of astrocytes in bacterial infections of the CNS by exploring their regulation of CNS inflammation, their ability to function as antigen presenting cells (APCs) and their interaction with T cells following microbial challenges.

**Table 1:** Astrocyte recognition of bacterial pathogens and immune mediator production.

Bacteria	PRR System	Cytokines	Chemokines	Clinical Significance	Reference
*Borrelia burgdorferi*	TLR-1, TLR-2, TLR-5, NOD-1, NOD-2	IL-1β, IL-6, IL-12, IL-23, TNF, IL-10 (cultures), IL-19	COX-2, CXCL-1, CXCL-10 IL-8	Lyme Neuroborreliosis	Constantinescu et al., 2005; Rasley et al., 2006; Chauhan et al., 2008, 2009; Lee et al., 2013; Cooley et al., 2014
*Brucella spp*	TLR-2	IL-1β, IL-6, TNF	CCL2 (MCP-1), CXCL1	Neurobrucellosis	Mosa et al., 2009; Lee et al., 2013
*Listeria monocytogenes*	TLR-2, NOD-1	Unknown	Unknown	Neonatal and adult meningitis	Mosa et al., 2009
*Mycobacteria tuberculosis*	Unknown	Unknown	CXCL10	CNS Tuberculosis	Rock et al., 2005
*Neisseria meningitidis*	NOD-2, SR-MARCO, Complement-CD46	IL-6, TNF IL-10 (cultures), IL-19	Unknown	Pediatric or Infant and adult meningitis	Rasley et al., 2006; Chauhan et al., 2009; Braun et al., 2011; Cooley et al., 2014
*Staphylococcus aureus*	TLR-2, NOD-2	IL-1β, IL-6, TNF	CCL2 (MCP-1), MIP-1β, and CXCL2 (MIP-2)	Brain abscesses and meningitis	Esen et al., 2004; Stenzel et al., 2008; Liu et al., 2010a
*Streptococcus agalactiae* (Group B Streptococcus)	Unknown	IL-1β, IL-6	IL-8	Neonatal meningitis	Stoner et al., 2015
*Streptococcus pneumoniae*	TLR-2, NOD-2	IL-19	Unknown	Neonatal, pediatric and adult meningitis	Liu et al., 2010b; Cooley et al., 2014


## Astrocytes in Pathogen Recognition

Under most circumstances bacterial pathogens invade the CNS via the bloodstream. Although precise mechanisms of entry into the CNS remains contested, it is now known that intracellular and extracellular bacteria evolved different strategies to circumvent host defense systems (Drevets et al., 2004). Once CNS barriers are breached, resident cells of the CNS recognize infectious non-self entities through a series of PRRs, which initiate a rapid immune response.

Toll like receptor are evolutionarily conserved type I membrane glycoproteins, comprising of eleven members in humans and thirteen members in mice, with discrete affiliations to specific ligands. Under physiological conditions TLRs are basally expressed in CNS areas lacking a BBB and therefore ideally positioned to interact with infiltrating pathogens in these areas. In human astrocytes, TLR3 is expressed constitutively at basal levels and significantly upregulated following treatment with IL-1β, IFN-β and IFN-γ (Farina et al., 2005; Jack et al., 2005). Additionally, TLR2, TLR4, TLR5 and TLR9 recognize bacterial ligands and are also expressed in resting astrocytes (Carpentier et al., 2005; Tarassishin et al., 2014). TLR2 recognition of bacterial peptidoglycan, lipopeptides and lipoprotein leads to astrocyte activation. For example, astrocyte activation combined with increased TLR2 expression was reported in the white matter of rhesus macaques infected with *Brucella melitensis* (Lee et al., 2013). TLR2 is also essential for the induction of cytokine and chemokine in astrocytes stimulated with *S. aureus* and peptidoglycan (Esen et al., 2004). Similar to TLR2, TLR4 signaling is required for protective immunity during CNS staphylococcal infection (Stenzel et al., 2008). In response to LPS *in vitro*, TLR4 activation in astrocytes generates MyD88-dependent NFκB signaling and subsequent upregulation of the target genes IL-15, IL-27, MMP-9, TNF, and VCAM-1 that may cause modifications of the BBB, prompt inflammation to recruit T lymphocytes and regulate immune responses (Gorina et al., 2011). LPS also causes delayed JAK1/STAT1 activation in astrocyte cultures, which was MyD88-independent and induced the expression of the negative cytokine regulator, SOCS-1 and chemokine CXCL10 (Gorina et al., 2011). These signals create a pro-inflammatory milieu, which regulate the activity of surrounding cells, facilitating microbial clearance. Both TLR2 and TLR4 are involved in the recognition of *Mycobacteria tuberculosis* (Ferwerda et al., 2005) and clearing *Brucella* spp infection from lungs (Lee et al., 2013). It is therefore possible that their induction in astrocytes may well point to a protective role during brain infection.

NOD1 and NOD2 proteins are part of NOD-like receptor family, that recognizes distinct motifs of intracellular pathogens. For instance, in addition to TLRs, the recognition of *M. tuberculosis* by NOD2 is important for activation of innate immunity (Ferwerda et al., 2005). In the context of CNS infection, NOD2 receptors function as intracellular sensors to *S. aureus*, *S. pneumoniae*, *B. burgdorferi* and *N. meningitidis*, which is upregulated in astrocytes (Chauhan et al., 2009; Liu et al., 2010a,b). Furthermore, the expression of NOD2 triggers the NFκB pathway via the adapter protein Rip2Kinase, ultimately inducing the production of IL-6 and TNF-α, and expression of co-stimulatory molecules which amplifies bacterially induced immune responses in astrocytes (Sterka et al., 2006).

Scavenger receptors (SRs), initially described as cell surface receptors on macrophages able to bind acetylated low-density lipoproteins, are now identified as a diverse group of PRRs that recognize various ligands including endogenous proteins and pathogens, participating in cell adhesion, phagocytosis and activation of immune responses. SR members, SR-MARCO and SR-A, also enable innate immune responses to Gram-negative and Gram-positive bacteria (Braun et al., 2011; Godoy et al., 2012; Dorrington et al., 2013). In human, MARCO variants are associated with increased susceptibility to pulmonary tuberculosis that may be attributed to its regulatory role in macrophage phagocytosis (Thuong et al., 2016). In murine astrocyte cultures, *N. meningitidis* and *S. pneumoniae* induce an upregulation of MARCO, which mediates the production of IL-1β in astrocytes (Braun et al., 2011). Therefore, its expression by astrocytes (Braun et al., 2011; Godoy et al., 2012) presents a compelling argument for a potential role in host defense during bacterial meningitis.

Complement is another important arm of innate immunity, known for its role in recognition and killing of pathogens including bacteria (Heesterbeek et al., 2018). Complement proteins are activated and found in the CSF of patients with bacterial meningitis (Shen et al., 2017). The functional role of complement components in CNS is further supported by the decreased survival of C1q and C3 deficient mice after induction of meningitis (Rupprecht et al., 2007). Notably, astrocytes can generate a majority of the complement components that can be modulated by various cytokines (Barnum et al., 1996). For example, LPS activated microglia release TNF and IL-1α, and in conjunction with C1q, induce A1 reactive astrocytes with elevated levels of C3 *in vitro* and *in vivo* (Liddelow et al., 2017; Clarke et al., 2018). It is therefore plausible that astrocyte dependent complement synthesis may have a significant role in regulating CNS immunity.

## Reactive Astrocytosis: Effects in Neuroinflammation and Neuroprotection

Under healthy conditions astrocytes maintain homeostasis and support neuronal survival through metabolic support (glutamate uptake and lactate export), ion homeostasis, neurotrophic factor release, synaptic maintenance and regulation of neurotransmitters, D-serine and purines (Kimelberg and Nedergaard, 2010; Zhang et al., 2014). However, following CNS insult such as injury and infection, astrocytes undergo a transformation process termed “reactive astrocytosis” during which the expression of multiple genes is altered (Liddelow et al., 2017; Clarke et al., 2018). The upregulation of glial fibrillary acidic protein (GFAP), the main cytoskeletal constituent of astrocytes, is a typical characteristic change in reactive astrocytosis and instrumental to control pathogenic spread (Stenzel et al., 2004). Another astrocytic protein, S100B, a calcium-binding protein, has also been used as a potential biomarker for CNS injury and diseases (Sen and Belli, 2007; Liddelow et al., 2017). S100B protein secretion can be induced by LPS administration in rats and in cultures (Guerra et al., 2011), and its physiological effect is dose-dependent. At high level, S100B is neurotoxic through its upregulation of iNOS and NO production *in vitro*, and promotes reactive astrocytosis *in vivo* (Hu et al., 1997; Villarreal et al., 2014). However, its extracellular activities remain further elucidation. Recent transcriptomic studies have characterized two subtypes of reactive astrocytes, namely A1 and A2, and accentuate the concept of reactive astrocytosis as a highly heterogenous state depending on the type of insult. While A2 reactive astrocytes are deemed to be neuroprotective through the release of neurotrophic factors which encourage CNS repair, the development of A1 reactive astrocytes is driven by LPS activated microglia and considered harmful by promoting neuroinflammation and neurotoxicity. These A1 astrocytes also loses their ability to support neuronal function and maintain synapses (Liddelow et al., 2017; Clarke et al., 2018). Astrocytes can also be directly stimulated by LPS or bacterial molecules to express various cytokines that includes IL-1β, IL-6, and TNF (Tarassishin et al., 2014), as well as several chemokines, CCL2, CXCL1, CCL20 and CCL3, suggesting that astrocytes modify the chemokine framework in a pathogen-specific manner (McKimmie and Graham, 2010). However, it is intriguing that human and mouse astrocytes have contrasting response to LPS which points to differential immune activation in species (Tarassishin et al., 2014). It is noteworthy that direct exposure of IL-1β to human fetal astrocyte cultures can induce reactive astrocytosis and change gene expression of inflammatory mediators, among which IL-6 and CXCL5 were prominently upregulated. Elevation of neurotrophic factor genes, such as BDNF and NGF, was also induced, suggesting that IL-1β may contribute to the neuroinflammatory and neuroprotective effects of human reactive astrocytosis (Teh et al., 2017).

TNF, a potent proinflammatory cytokines, is accepted as a principle cytokine involved in antimicrobial Th1 immunity. Studies using mice lacking TNF have shown that it is critical to control cerebral tuberculosis (Hsu et al., 2017). TNF is also detected in the CSF of Lyme neuroborreliosis patients and the brains of the *B. burdorferi* infected mice (Chauhan et al., 2008). The production of TNF together with IFN-γ, IL-12, IL-23, and NO by astrocytes supports their potential involvement in host immunity to CNS tuberculosis (Constantinescu et al., 2005; McKimmie and Graham, 2010; Tarassishin et al., 2014). Astrocytes enhance TNF and IL-12 concentrations in the brain during microbial invasion, and their increased reactivity in the presence of TNF and IFN-γ, insinuates their potential contribution to these pathways in CNS host defense. Furthermore, astrocytes may mediate neuroprotection through the release of neurotrophic factors after stimulation with TNF and LPS *in vitro* (Saha et al., 2006). After LPS treatment one in particular namely astrocyte IL-6 enhances neuronal survival in co-cultures (Sun et al., 2017).

Similar to TNF, IL-6 is a pleiotropic cytokine, and its classical pathway involves the binding of IL-6 to IL-6R and gp130 that subsequently activate JAK/STAT signaling. The upregulation of IL-6 is common to many CNS bacterial infections (Chauhan et al., 2008; Stenzel et al., 2008; Stoner et al., 2015), and in the CSF of meningitis patients (Misra et al., 2010; Pinto Junior et al., 2011). Given that, IL-6 deficient mice fail to control *M. tuberculosis* infection (Ladel et al., 1997), IL-6 is required for immune protection. Moreover, in GFAP-IL6 transgenic mice, the over-expression of IL-6 in astrocytes was sufficient to induce reactive astrocytosis. Interestingly, the use of gp130 to block IL-6 can reduce astrocytosis in GFAP-IL6/sgp130 mice (Campbell et al., 2014). These findings suggest that chronic expression of IL-6 by astrocytes has a critical role in neuropathological effects during CNS immune responses.

Delayed microglial and astrocytic production of immunosuppressive cytokines, such as IL-10 is generated in response to *B. burgdorferi* and *N. meningitidis*, suggesting the possibility of negative feedback loops whereby mediators act to limit potentially damaging inflammation within the CNS during chronic infections (Rasley et al., 2006). Interestingly, the production of IL-10 in the mouse brain was significantly reduced following *in vivo* infection of *B. burgdorferi* or *N. meningitidis*, while the levels of both IL-6 and TNF-α were significantly elevated (Chauhan et al., 2008). IL-10 influences many aspects of immune responses and is an effective inhibitor of activated glia through suppression of their proinflammatory cytokine response pathways (Rasley et al., 2006). Expression of IL-19 is also induced in the brains of mice as well as astrocyte cultures following bacterial challenge. Astrocyte treatment with IL-19 stimulated upregulation of SOCS3, which inhibits cytokine synthesis. As a result, it also reduced astroglial production of IL-6 and TNF following bacterial challenge (Cooley et al., 2014). Therefore, the induction and modulation of proinflammatory cytokines and immunosuppressive cytokines support the regulatory role of astrocytes to maintain a delicate balance through promoting cellular responses and limiting inflammation following CNS infection.

## Astrocyte-T Cell Interaction

**FIGURE 1:**
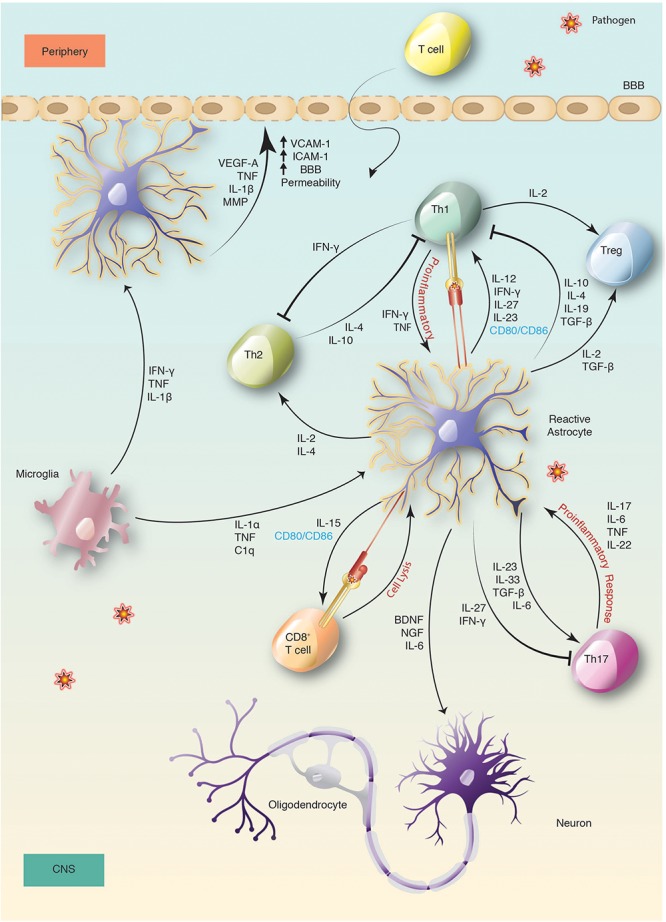
Activation and immune regulation of astrocyte in bacterial infection. Activation of microglia and astrocytes is a sensitive indication of changes following CNS infection. This leads to secretion of cytokines and chemokines by reactive astrocytes that changes the BBB permeability, mediates neuroprotection and promotes the recruitment of peripheral immune cells, such as T cells. Recruited T cells then infiltrate into the CNS parenchyma across the BBB and after entering, are restimulated by reactive astrocytes.

Communication between the CNS and peripheral immune system through a lymphatic system within the meninges indicates that the CNS is under constant immune surveillance (Louveau et al., 2015). Patrolling APCs within this system would proceed to the cervical lymph nodes after an encounter with pathogens that invaded the CNS, and prime naïve T cells for maturation. Activated T cells comfortably cross the BBB with the help of ICAM-1 and/or VCAM-1 (Gimenez et al., 2004). These adhesion molecules facilitate astrocyte-lymphocyte interaction which are upregulated upon astrocyte exposure to IFN-γ, TNF-α, IL-1β or TLR ligands such as LPS *in vitro* (Carpentier et al., 2005). Recruited T cells then infiltrate into the CNS parenchyma and are restimulated by native APCs such as astrocytes and microglia (Figure 1).

Expression of MHC-II molecules is critical to initiate immune responses by presenting processed antigen to CD4^+^ T-helper cells via the T cell receptor (TCR). While both glial cell types, astrocytes and microglia, induce MHC-II and co-stimulatory signals under inflammatory conditions to activate T cells (Constantinescu et al., 2005), the role of astrocytes in presenting antigen is contentious. IFN-γ is efficient at inducing MHC II expression on microglia, but its effects on astrocytes may be delayed (Esen et al., 2004). Studies indicate that IFN-γ and TNF-treated astrocytes upregulate the expression of MHC-II, B7-1 and B7-2, for antigen presentation and proficiently activate Th1 or Th2 CD4^+^ T cells (Gimsa et al., 2004; Carpentier et al., 2005). Others showed that IFN-γ-activated astrocytes are more efficient at stimulating Th2 rather than Th1 cells, which leads to IL-4 expression (Aloisi et al., 1998). APCs and phagocytic cells produce IL-12, which is important for T-cell priming and the development of Th1 type immune responses. IL-23, a member of the IL-12 family, is also necessary for the establishment of T-cell mediated inflammation and involved in both Th1 and Th17 responses (Constantinescu et al., 2005). Under inflammatory conditions, astrocytes express IL-12 and IL-23, and present antigen to encephalitogenic T-cells in an IL-12/IL-23 dependent manner (Constantinescu et al., 2005). Together with IL-12, IFN-γ drives T-cell maturation toward a Th-1 phenotype. Astrocyte expression of IL-4 and IL-10, reduces Th1 cytokine expression and induces naïve T cells to differentiate into Th2 cells (Meeuwsen et al., 2003), indicative of a regulatory role in the Th1/Th2 balance of the CNS. Alternatively, astrocytes may participate in neuroprotection by upregulating CTLA-4 to reduce T-cell responses in an effort to impede inflammation in the CNS (Gimsa et al., 2004). Although T cell-astrocyte interaction *in vivo* is not well documented, it is a noteworthy association with important implications in the control of neuroinflammation.

Distinct from the Th1 or Th2 lineage is the Th17 effector CD4^+^ T cells, also activated by IL-23, but which occurs in the presence of TGF-β and IL-6 (Bettelli et al., 2006; Mangan et al., 2006). TGF-β is predominantly associated with immune-suppression via Th1 and Th2 inhibition as well as the development of T-regulatory cells (Bettelli et al., 2006). However, it is now apparent that TGF-β is crucial for Th17 progression. TGF-β signaling in an inflammatory climate aids IL-23 recognition by upregulating IL-23R expression, thus promoting Th17 development (Mangan et al., 2006). Interestingly, by transferring Th17 supernatants to astrocyte cultures, Th17 effector molecules could directly influence astrocytic function and phenotype (Prajeeth et al., 2017). IL-17 in particular can induce IL-6 production by astrocytes that triggers a positive-feedback loop of IL-6 to promote Th17 cell differentiation. Thus, the release of IL-6 by astrocytes is a proficient mechanism to facilitate Th17 development (Ma et al., 2010). The correlative and inimical action of astrocytic cytokines regulates Th1 and Th17 polarization, indirectly coordinating the cytokine reservoir, and ultimately influencing the adaptive immune response. In this way astrocytes help equip the CNS to combat permeating pathogens through expansion of the relevant T cells.

## Conclusion and Future Outlook

Astrocytes play a progressive role in maintaining CNS homeostasis, in both physiological and pathological conditions, such as bacterial infection. Astrocytes located in different parts of the brain have functional differences, but whether these subpopulations also exhibit differences in susceptibility to infection, inflammatory responses or effects on BBB function remains to be determined. They are actively involved in host defense and immunity, and their proclivity toward driving the development and/or recruitment of a particular cell type is largely regulated by local prevailing immune conditions. Astrocytes have been identified as heterogenous cells, and their response is ultimately dictated by the insult which determines their influence on other cell types and their mediation of secondary immune responses through the expression of cell surface markers and soluble factors. Their phenotype may be protective or causative regarding neuropathology. Unfortunately, the threat of mortality and substantial neurological complications associated with CNS bacterial infection is greater in developing countries due to the lack of efficient diagnosis and treatment. There is a need for the development of targeted therapy against CNS infection. By identifying the mechanisms that drive astrocyte diversity in CNS bacterial infection, strategies for intervention can be developed for enhanced treatment and improved prognosis.

## Author Contributions

SG and N-JH wrote the manuscript. SG prepared the figures. MJ critically revised the manuscript.

## Conflict of Interest Statement

The authors declare that the research was conducted in the absence of any commercial or financial relationships that could be construed as a potential conflict of interest.

## Abbreviations

CNS: central nervous system
CSF: cerebrospinal fluid
ICAM: intracellular adhesion molecule
IFN: interferon
IL: interleukin
LPS: lipopolysaccharide
MCP: monocyte chemotactic protein
MHC: major histocompatibility complex
MMP: matrix metallometalprotease
PRR: pattern recognition receptor
TLR: toll like receptor
TNF: Tumor necrosis factor
